# Functional and structural characteristics of anticancer peptide Pep27 analogues

**DOI:** 10.1186/1475-2867-5-21

**Published:** 2005-07-11

**Authors:** Dong Gun Lee, Kyung-Soo Hahm, Yoonkyung Park, Hai-Young Kim, Weontae Lee, Sung-Chul Lim, Youn-Kyung Seo, Cheol-Hee Choi

**Affiliations:** 1 School of Life Science and Biotechnology, College of Natural Sciences, Kyungpook National University, 1370 Sankyuk-dong, Puk-ku, Taegu 702-701, Korea; 2 Research Center for Proteinous Materials, Chosun University, Gwangju 501–759, Korea; 3 Departement of Biochemistry and HTSD-NMR National Research Laboratory, Yonsei University, Seoul, South Korea; 4 Department of Pathology, College of Medicine, Chosun University, Gwangju 501–759, Korea; 5 Research Center for Resistant Cells and Department of Pharmacology, College of Medicine, Chosun University, Gwangju 501–759, Korea

**Keywords:** Peptide Pep27, Pep27 analogues, *S. pneumoniae*, apoptosis, anticancer activity, 3D structure

## Abstract

**Background:**

A secreted peptide Pep27 initiates the cell death program in *S. pneumoniae *through signal transduction. This study was undertaken to evaluate the relation between the structure and cytotoxic activity of Pep27 and its analogues on cancer cells.

**Results:**

Pep27anal2 characterized substituting (^2^R→W), (^4^E→W), (^11^S→W) and (^13^Q→W) in native Pep27, exhibited greater hydrophobicity and anticancer activity than Pep27 and other analogues. The IC_50 _values of Pep27anal2 were approximately 10 – 30 μM in a number of cell lines (AML-2, HL-60, Jurkat, MCF-7 and SNU-601). Confocal microscopy showed that Pep27anal2-FITC was localized in the plasma membrane, and then moving from the membrane to subcellular compartments with the initiation of membrane blebbing. Flow cytometric analysis using propidium iodide and Annexin V also revealed that Pep27anal2 induced apoptosis with minor membrane damage. Electron microscopy revealed that Pep27 induced apoptosis in Jurkat cells. The anticancer activity of Pep27anal2 was neither abrogated by pan-caspase inhibitor (Z-VAD-fmk) nor related to cytochrome c release from mitochondria. The 3D solution structures of these two Pep27 peptides revealed that both form a random coil conformation in water; however, they adopted stable α-helical conformations in solutions.

**Conclusion:**

The results indicate that Pep27anal2 can penetrate the plasma membrane, and then induce apoptosis in both caspase-and cytochrome c-independent manner. The hydrophobicity of Pep27anal2 appears to play an important role in membrane permeabilization and/or anticancer properties. The structure-functional relationships of these peptides are also discussed. It is proposed that Pep27anal2 is a potential candidate for anticancer therapeutic agents.

## Introduction

Newly discovered peptides are becoming increasingly important, not only as molecular tools for the understanding of protein-protein interactions, but also as lead compounds. Cationic amphipathic peptides, such as cecropins, defensins, and mellitin, induce cell death in prokaryotic and eukaryotic cells by increasing membrane permeability [1]. This increased permeability may lead to cell lysis or, alternatively, may produce subtle changes in the membrane barrier function and promote cell death [2]. It has also been shown that the anticancer activities of natural and synthetic cationic peptides exhibit therapeutic activity in murine models of human tumors [3-5].

It has been proposed that processes occurring within bacteria are necessary to trigger endogenous suicidal enzymes that dissolve the cell wall during antibiotic-induced autolysis. Recently, Pep27, was found to initiate the cell death program in *S. pneumoniae *through signal transduction triggered via the two-component system (VncR/S) consisting of a membrane-bound histidine protein kinase (e.g., VncS) and a cytoplasmic effector termed a response regulator (e.g., VncR) [6]. Pep27 is secreted through the Vex ABC transporter and accumulates in the medium [6,7]. Accumulated Pep27 is then sensed by VncS, converting this histidine protein kinase into a phosphatase that dephosphorylates phosphorylated VncR, allowing derepression of autolytic pathways and resulting in the loss of viability and lysis of pneumococcal cells.

The present study was undertaken to identify analogues of bacterial death signal peptide Pep27 capable of inhibiting the growth of cancer cells, and to investigate the nature of the relation between their biologic behaviors and their molecular structures.

## Materials and methods

### Culture

OCI-AML-2 (AML-2) cell line was obtained from the Ontario Cancer Institute (Toronto, Canada), and Jurkart and HL-60 cell lines from the American Type Culture Collection (ATCC, USA). These three cell lines were cultured at 37°C in a 5% CO_2 _atmosphere using α-MEM medium (GibcoBRL, Gland Island, NY, USA) containing 10% heat-inactivated fetal bovine serum (FBS, Sigma, ST. Louis, MO, USA). MCF-7 and NIH/3T3 cell lines from ATCC and SNU-601 cell line from the Korean Cancer Cell Bank (Seoul, Korea) were cultured under the same conditions but using RPMI-1640 medium (GibcoBRL, Gland Island, NY, USA) containing 10% heat-inactivated FBS. Cells were maintained in suspension or as monolayer cultures, and subcultured.

### Peptide synthesis

Peptides were synthesized by using a solid phase method using (based on) Fmoc (9-fluorenyl-methoxycarbonyl)-chemistry [8]. Rink amide 4-methyl benzhydrylamine (MBHA) resin (0.55 mmol/g) was used as the support. Coupling of Fmoc-amino acids was performed by DCC (dicyclohexyl-carbodiimide/HOBt (1-hydroxybenzotriazole). After completion of peptide chain elongation, the protected final peptide resins were treated with the reagent K. The crude peptide obtained was then washed repeatedly with diethylether, dried in vacuum, and purified by reverse-phase (RP) HPLC (LC10A, Shimadzu, Tokyo, Japan) on a Waters 15-m Deltapak C18 column. The purity of the peptide was checked by analytical RP-HPLC using an Ultrasphere C18 column (4.625 cm, Beckman, Miami, FL, USA). Purified peptides were hydrolyzed with 6 N HCl at 110°C for 22 h, and then dried in vacuum. The residues were dissolved in 0.02 N HCl and subjected to amino acid analysis (8500 A, Hitachi, Tokyo, Japan) to determine peptide concentration. The molecular weights of the synthetic peptides were determined by MALDI (matrix-assisted laser desorption ionization) mass spectra, respectively (data not shown).

### Cytotoxicity test

The *in vitro *cytotoxicity of the Pep27 analogues produced was measured by MTT [3-(4,5-dimethylthiazol-2-yl)-2,5-diphenyl tetrazolium bromide, Sigma, ST. Louis, USA] assay, as described by [9]. IC_50 _values were determined directly from semilogarithmic dose-response curves. Experiments were carried out at least in duplicate.

### Hemolytic activity

Peptide hemolytic activities were measured as described by [10], by determining hemoglobin release by 4% suspensions of fresh human erythrocytes at 414 nm. Briefly, human erythrocyte cells were centrifuged and washed three times with phosphate-buffered saline (PBS: 35 mM phosphate buffer/0.15 M NaCl, pH 7.0). 100 ml of human red blood cells suspended 8% (v/v) in PBS were the inoculated into 96-well plates. 100 ml of the peptide solution, Pep27 or its analogues, was then added to each well. The plates were incubated for 1 h at 37°C, and centrifuged at 150 g for 5 min. 100 ml aliquots of the supernatant obtained were transferred to flat-bottom 96-microtiter plates. Hemolysis was determined by measuring absorbance at 414 nm with an ELISA plate reader (Molecular Devices Emax, Sunnyvale, CA, USA). Hemolysis rates of 0% or 100% were determined as the same way with PBS and 0.1% Triton-100, respectively. Hemolysis percent was calculated using the following equation: % hemolysis = [(Abs414nm in the peptide solution - Abs414nm in PBS)/(Abs414nm in 0.1% Triton-100 - Abs414nm in PBS)] × 100.

### Preparation of fluorescein isothiocyanate (FITC)-labeled peptide

FITC was freshly dissolved in DMSO to 10 mg/ml, and added to 1 mg/ml of peptide in 100 mM sodium bicarbonate, pH 9.3 to a final concentration of 1 mg/ml FITC. After incubation for 4 hr in the dark at room temperature, 1 M ethanolamine was added to inactivate the residual FITC. The solution was then left in the dark for an additional 2 hr, and chromatographed sequentially on a gel-filtration column followed by a 120 cm Sephadex G-50 column to remove unconjugated dye. The FITC-labeled peptide (FITC-Pep27anal2) was obtained by reverse phase HPLC using a C18 column. Conjugation between FITC and the peptide of interest was confirmed by SDS-PAGE gel as intense peptide fluorescence under UV light.

### Confocal microscopy

Jurkat cells grown on glass coverslips overnight were treated with FITC-labeled Pep27anal2 at a concentration of 5 μg/ml. The cells were washed with PBS at 30, 60 and 120 min being treated of Pep27anal2, and then fixed using 3.7% formaldehyde for 15 min. Coverslips were mounted on glass slides, FITC was excited using a 488-nm laser, and images were manipulated using the manufacture's software.

### Determination of apoptosis and membrane integrity

Annexin V binding was assessed by flow cytometry, and cell staining was evaluated using FITC-labeled annexin V (green fluorescence), and propidium iodide (PI) (negative for red fluorescence) simultaneously. This test was able to discriminate between intact cells (FITC-/PI-), apoptotic cells (FITC+/PI-) and necrotic cells (FITC+/PI+) (Vermes et al., 1995). Jurkat cells (10^5^/ml) were exposed to etoposide (100 μM), mellitin (12.5 μM) and Pep27anal2 (12.5 μM and 62.5 μM) for 4 hr. Cells were washed with PBS, resuspended in 400 μl of binding buffer (10 mM Hepes/NaOH, pH 7.4, 140 mM NaCl, 2.5.mM CaCl2), and incubated with 10 μl of annexin V-FITC (MedSystems Diagnostics, GmbH, Australia) for 10 min at room temperature. After incubation, cells were washed with binding buffer and exposed to 1 μg of PI in a final volume of 400 μl. FITC and PI fluorescences were determined using a flow cytometer (Becton Dickinson, Franklin Lakes, FL, USA)

### Electron microscopy

Jurkat cells were harvested and immediately fixed in phosphate-buffered glutaraldehyde solution (pH 7.2) for 2 hrs and then fixed in 1% buffered osmium tetroxide for 2 hrs at 4°C. After processing through a graded series of ethanol and propylene oxide, the cells were embedded in Epon. A semi-thin (1 μm) section was cut and stained with toluidine blue for the ultrastructural examination. Ultra-thin (60–90 nm) sections were also prepared from the Epon block above using an ultramicrotome (LKB-V, Sweden), stained with uranyl acetate and lead citrate, and examined under a Hitachi H-7600 electron microscope (Hitachi, Kyoto, Japan). Apoptosis detection was done by taking photographs (×1,000) of the entire field of each ultra-thin sectioned sample. The degree of apoptosis (apoptosis index) was defined as the number of apoptotic cells expressed as a percentage of the total cell count.

### Preparation of cytosolic extracts and the immunoblotting of cytochrome c

Cytochrome c immunoblotting was performed using a slight modification of a previously described method [11]. Jurkat cells were collected by centrifugation at 200 g for 5 min at 4°C. The cells were then washed twice with ice-cold PBS, pH 7.4, and centrifuged at 200 g for 5 min. The cell pellet obtained was then resuspended in 600 μl of extraction buffer, containing 20 mM Hepes-KOH (pH 7.5), 10 mM KCl, 250 mM sucrose, 1 mM EGTA, 1 mM EDTA, 1.5 mM MgCl_2_, 1 mM dithiothreitol and 0.1 mM phenylmethylsulfonyl fluoride.

After incubation for 30 min on ice, the cells were homogenized (20 strokes), spun at 14,000 g for 15 min, supernatants were separated from the mitochondrial fraction, and both were stored at -70°C, until required for gel electrophoresis. The mitosol fraction was obtained by adding 0.1% SDS. Fifty μg of cytosolic protein extracts and the mitosol extracts were loaded into the lanes of an 15% SDS-polyacrylamide gel, separated and blotted onto a nitrocellulose membrane (Amersham, Piscataway, NJ, USA) at 150 mA overnight in transfer buffer (20 mM Tris-base, 150 mM glycine, 20% methanol). Non-specific binding was blocked by incubating the membrane in 5% non-fat milk in TBS-T (Tris-buffered saline and Tween-20; 10 mM Tris-HCl pH 8.0, 50 mM NaCl, and 0.05% Tween-20) for 3 h at RT. The membrane was incubated in anti-cytochrome c monoclonal antibody (7H8.2Cl2) solution (1:2000 dilution, BD PharMingen, San Diego, CA, USA) containing 0.5% non-fat milk in TBS-T. After overnight incubation with agitation at 4°C, the membrane was washed three times with 0.5% non-fat milk in TBS-T. The secondary antibody, a horseradish peroxidase-coupled anti-mouse antibody (Sigma, ST. Louis, MO, USA), was incubated at a 1:1000 dilution for 2 h at RT. The membrane was then washed four times (5 min) with 5% non-fat milk in TBS-T.

### NMR (nuclear magnetic resonance) spectroscopy

The peptide samples used for NMR experiments contained 1.5 mM peptide in water /2, 2, 2-trifluoro-(*d*_3_)-ethanol (TFE) mixture (50:50; v/v) at pH 7.2, which was prepared after lyophilizing samples in an aqueous solution. NMR spectra were recorded at 15°C on a Bruker DRX-500 spectrometer equipped with a triple-resonance probe and an x, y, z-shielded pulsed-field gradient coil. Two-dimensional (2D) NMR spectra were recorded in the phase-sensitive mode using time-proportional phase incrementation [12] for quadrature detection in the *t*_1 _domain. The 2D experiments such as TOCSY was performed using a MLEV-17 spin-lock pulse sequence with a mixing time of 69.7 ms and 2D-NOESY with mixing time of 300 ms. Solvent suppression during TOCSY and NOESY experiments was achieved using a WATERGATE pulse sequence [13] in combination with a pulsed-field gradient pulse. All NMR spectra were acquired with 2048 complex the *t*_2 _data points and 256 increments in the *t*_1 _dimension, with 32 scans per increment. Slowly exchanging amide protons were identified by lyophilization of a fully protonated sample in 50% H_2_O/ 50% TFE, redissolution in 50% D_2_O/ 50% TFE solution, and the immediate acquisition of a series of one-dimensional NOESY and 2D-NOESY spectra. _3_*J*_HNα _coupling constants were measured from the 1D spectrum. NMR data were processed on a Silicon Graphics Indigo II workstation using XWINNMR (Bruker Instruments, Karlsruhe, Germany) software. Proton chemical shifts were expressed relative to the methyl resonance of sodium 2, 2-dimethyl-2-silapentane-5-sulfonic acid (DSS), used as an internal standard.

### Statistical analysis

Data are expressed as means ± SEM. One-way ANOVA and the Student's *t*-test were performed using the Statistical Package for the Social Sciences (SPSS) for Windows, version 7.5 (SPSS, Korea). Results were considered significant when *p *was <0.05.

## Results

### Design and synthesis of peptides

The amphipathic features of α-helical antimicrobial peptides plays an important role against target cells. Furthermore, a number of parameters, including a net positive charge, α-helicity, and overall hydrophobicity have been shown to modulate the antibiotic activity of the α-helical amphipathic antimicrobial peptides. Recent investigations using amphipathic α-helical model peptides have revealed that the modulation of the hydrophilic-hydrophobic balance of α-helical antimicrobial peptides is a crucial factor in the design of peptides with potent antibiotic activity [14]. In a preliminary experiment, we could not find that Pep27 has anticancer activity. Therefore, we designed Pep27 analogue peptides and evaluated their anticancer and chemosensitizing effects. In order to elucidate the relationship between the hydrophobic and the hydrophilic regions of antibiotic peptides with respect to antibiotic activity and cytotoxicity, we designed and synthesized certain Pep27 analogues, on the basis of the α-helical wheel diagram of Pep27. In particular, Pep27 hydrophobicity was increased by substituting tryptophan. The amino acid sequences used in the present study are summarized in Table 1. Synthesized peptides were purified by RP-HPLC and their molecular weights were confirmed by MALDI-MS.

**Table 1:** Amino acid sequences of Pep27 derived from *S. pneumoniae *and its analogue peptides, and their retention times

**Peptide**	**Amino acid sequences**	**Remarks (substitution)**	**RT (min)**
Pep27	MRKEFHNVLSSGQLLADKRPARDYNRK	(^2^R→W), (^4E^→W)	14.54
Pep27anal1	MWKWFHNVLSSWQLLADKRPARDYNRK	(^2^R→W), (^4^E→W)	18.67
Pep27anal2	MWKWFHNVLSWWWLLADKRPARDYNRK	(^2^R→W), (^4^E→W), (^11^S→W), (^13^Q→W)	22.50
Pep27anal3	MRKWFHNVLSSGQLLADKWPAWDYNRK	(^4^E→W), (^19^R→W), (^22^R→W), (^26^R→W)	20.08
Pep27anal4	MWKEFHNVLSSGQLLADKRWARWYNRW	(^2^R→W), (^20^P→W), (^23^D→W), (^27^K→W)	19.15
Pep27anal5	MWKWFHNVLSSGQLLADKWWAWWYNWW	(^2^R→W), (^4^E→W), (^19^R→W), (^20^P→W), (^22^R→W), (^23^D→W), (^26^R→W), (^27^K→W)	19.17

RT, retention time obtained by RP-HPLC

RP-HPLC has often been used to experimentally determine the quantitative hydrophobic-hydrophilic balance of amphipathic peptides. Peptides are separated by RP-HPLC according to their hydrophobic interactions with C-18 of the stationary phase. Such hydrophobic interactions can be considered comparable to the interaction between amphipathic antimicrobial peptides and the lipid bilayer of plasma membranes. In order to investigate the possibility of a correlation between peptide hydrophobicity and the biological activities on cancer cells, the hydrophobic characteristics of the peptides were investigated by comparing their retention times on RP-HPLC (Table 1). As expected, all Pep27 analogues eluted later than Pep27, and the analogue Pep27anal2 had the longest retention time.

### Anticancer activities of Pep27 and its analogues

**Figure 1:**
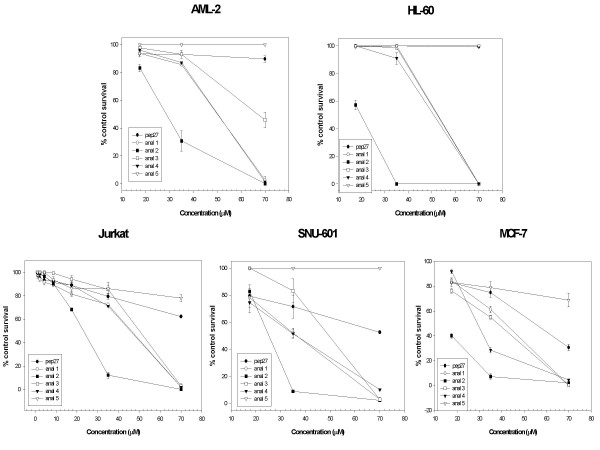
**Cytotoxicity of Pep27 and of its analogues in various cancer cell lines by MTT assay. **AML-2, human acute myelogenous leukemia cell line; HL-60, human promyelocytic leukemia cell line; Jurkat, T-cell leukemia cell line; SNU-601, human gastric adenocarcinoma cell line; MCF-7, human breast cancer cell line

To evaluate the anticancer activities of the Pep27 analogues, different concentrations of these peptides were administered to various cancer cell lines, and cytotoxicities were then determined by MTT assay. In this study, we used both anchorage-dependent and independent cancer cell lines. These included AML-2 an acute myelogenous leukemia cell line, HL-60 an acute promyelocytic leukemia cell line, Jurkat a T cell leukemia cell line, SNU-601 a gastric cancer cell line and MCF-7 a breast cancer cell line. Pep27 analogue peptides showed similar anticancer activities in five cancer cell lines tested. Of the analogues tested, Pep27anal2 showed most anticancer activity (Fig. 1). The IC_50 _and IC_90 _values of Pep27anal2 in five cancer cell lines tested were 10 – 28 μM and 35 – 55 μM, respectively (Fig. 1 and Table 2). Since the substitution of (^2^R→W) and (^4^E→W) to form Pep27anal1 showed less activity than Pep27anal2, the additional substitution of (^11^S→W) and (^13^Q→W) in Pep27anal2 was viewed as being important for anticancer activity. On the other hand, other Pep27 analogues substituted with more than two tryptophans at different sites did not show more anticancer activity than Pep27anal2 in 5 cell lines tested. Moreover, of the Pep27 analogues produced, Pep27anal2 had the greatest hydrophobicity, based on its RP-HPLC retention time (Table 1).

**Table 2:** Anticancer activity of Pep27 and of its analogues in various cancer cell lines by MTT assay

**Peptide**	**IC_50 _(μM)**				
	
	**AML-2**	**HL-60**	**Jurkat**	**SNU-601**	**MCF-7**
Pep27	>70	>70	>70	>70	>70
Pep27anal1	50	53	47	37	55
Pep27anal2	29	20	23	25	<10
Pep27anal3	67	52	50	50	38
Pep27anal4	50	51	46	37	29
Pep27anal5	>70	>70	>70	>70	>70

AML-2, acute myelogenous leukemia cell line; HL-60, acute promyelocytic leukemia cell line; Jurkat, T cell leukemia cell line; SNU-601, gastric cancer cell line; MCF-7, breast cancer cell line

### How does Pep27anal2 show its cytotoxic effect in cancer cells?

**Figure 2:**
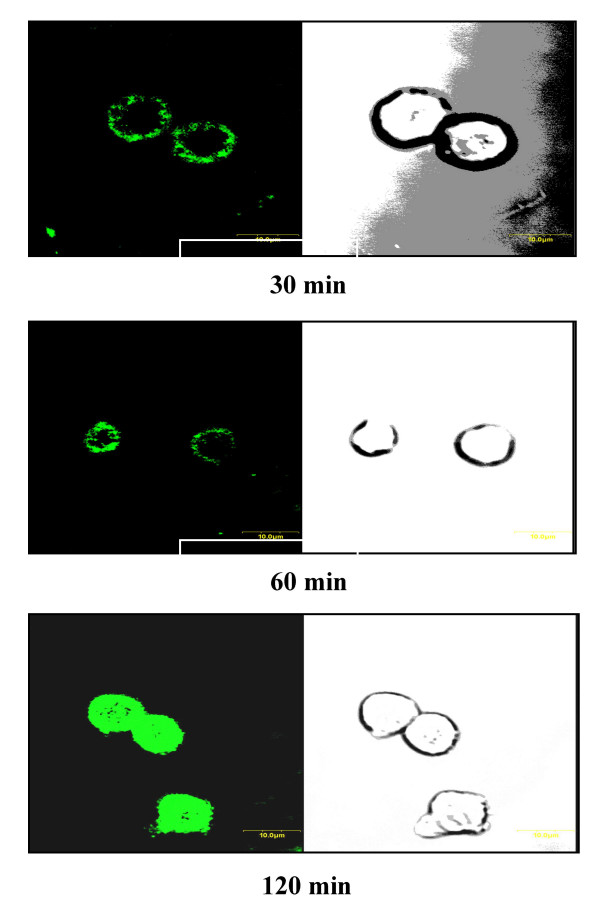
**Confocal microscopy of Jurkat cells after treatment with FITC-labeled Pep27anal2 as a function of time. **After incubating FITC-labeled Pep27anal2 for 30, 60, and 120 min, cells were fixed with 3.7% formaldehyde and analyzed using a laser scanning confocal microscope. Pep27anal2 peptide expression is shown as green on the left confocal microphotograph. Cells morphology is shown by the phase contrast microphotograph on the right.

In order to determine whether or not Pep27anal2 can cause membrane damage, an RBC hemolysis test was performed (Table 3). Of the Pep27 analogues, only Pep27anal 2 at 12.5 μM for 1 h at 37°C, hemolysed 18% of RBCs whereas mellitin at the same concentration induced 100% hemolysis, suggesting that Pep27anal2 may cause membrane damage like mellitin. Since the mechanism of Pep27anal 2 peptides is believed to involve disruption of the cell membrane, confocal microscopy used to determine whether Pep27anal2 can enter cells and act as a membrane toxin. Jurkat cells were treated with Pep27anal2-FITC at a concentration of 12.5 μM for 30, 60 and 120 min. After incubation cells were fixed with 3.7% formalin for 30 min and then observed under a confocal microscope. Confocal microscopy showed ring-shaped cells, suggesting that Pep27anal2-FITC is localized to the cell membrane without disturbing its integrity, and then moves from the membrane intracellularly with time (Fig. 2). However, Jurkat cells treated with 12.5 μM Pep27anal2 for more than 60 min showed cell shrinkage and membrane blebbing, suggesting apoptosis, and indicating that Pep27anal2 can enter cells by an unknown mechanism and induce apoptosis whilst maintaining membrane integrity, within 2 hr of treatment.

**Table 3:** Hemolytic activity of Pep27 and its analogues

**Peptide**	**% Hemolysis**						
	
	**0.19 μM (μM)**	**0.39 μM**	**0.78 μM**	**1.56 μM**	**3.12 μM**	**6.25 μM**	**12.5 μM**
Pep27	0	0	0	0	0	0	0
Pep27anal1	0	0	0	0	0	0	0
Pep27anal2	0	0	0	0	0	0	18
Pep27anal3	0	0	0	0	0	0	0
Pep27anal4	0	0	0	0	0	0	0
Pep27anal5	0	0	0	0	0	0	0
Mellitin*	0	0	12	30	60	86	100

*, honey bee venom, which translocates across the membrane by forming a pore.

### Determination of apoptosis after treating with Pep27anal2

One of the earliest events to occur during apoptosis is the externalization of phosphatidylserine, a phospholipid that is normally restricted the inner leaflet of the plasma membrane. Importantly, this precedes membrane damage. Moreover, the externalization of phosphatidylserine can be monitored using annexin V, a phosphatidylserine-specific binding protein [15]. Another important secondary event during apoptosis is the loss of plasma membrane integrity. Intact plasma membranes exclude certain dyes, like PI, the most commonly used marker of membrane integrity [16].

**Figure 3:**
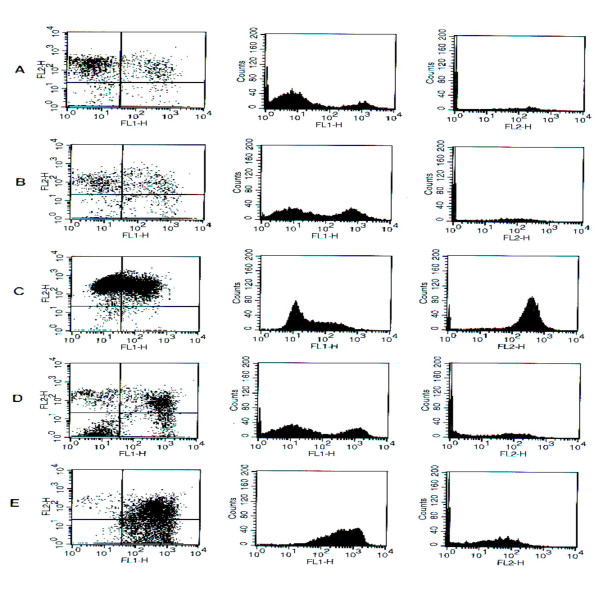
**Determination of apoptosis and membrane integrity by FACS. **Cells were treated with etoposide, mellitin and Pep27 anal2. Apoptosis and membrane integrity were determined using annexin V and propidium iodide (PI), respectively, as described in Materials and Methods. A, control; B, 100 μM etoposide; C, 12.5 μM mellitin; D, 12.5 μM Pep27anal2; E, 62.5 μM Pep27anal2. Left, dual staining for annexin V-FITC (FL1-H) and PI (FL2-H); Middle, distribution of annexin V-FITC staining in cell populations; Right, distribution of PI staining. Similar results were obtained in independent duplicated experiments.

**Figure 4:**
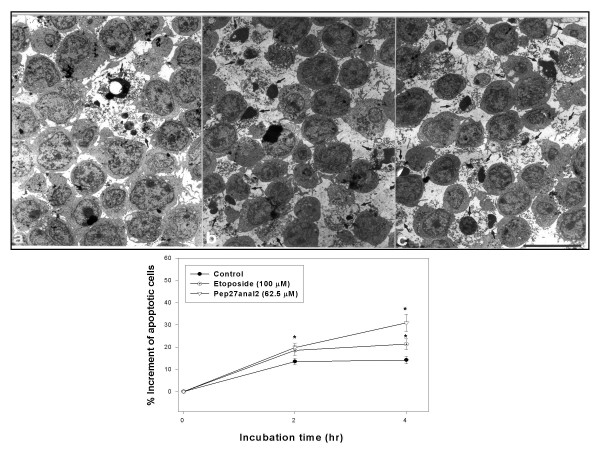
**Time-dependency on Pep27anal2 – induced apoptosis in Jukart cells**. Apoptotic cells were counted among 200–400 cells in each group. The representative electron microscopic findings show apoptosis induced by etoposide or by Pep27anal2 after 4-hr incubation. (a) Control group. Some apoptotic cells were found. (b) Etoposide treated group. Apoptotic cells were more frequent than in the control group. (c) Pep27anal2 treated group. Apoptotic cells were most frequent in this group. Lead citrate and uranyl acetate staining. Scale bar measures 9.9 μm. Arrows indicate apoptotic cells.

In the present study, Jurkat cells were treated with 100 μM of etoposide, 12.5 μM of mellitin or 12.5 μM or 63 μM of Pep27anal2 for 4 hr. Apoptosis and membrane integrity were then determined using annexin V and propidium iodide, respectively, as described in "Materials and methods". In Fig. 4. living cells are confined to the lower left quadrant. Dead cells appear first in the lower right quadrant (apoptosis), where death was due to the externalization of PS (detected with annexin V-FITC). The upper right quadrant shows cell death resulting from the loss of cell membrane integrity (PI incorporation). Fig. 3 shows that Pep27anal2 caused a higher level of annexin V-FITC staining with a little increase of PI staining than etoposide, whereas mellitin increased the distribution of PI staining without an increase of annexin V-FITC staining.

An electron microscopic examination revealed that Pep27anal2 induced apoptosis in Jurkat cells, as characterized by condensation of the cytoplasm, cell shrinkage, loss of plasma membrane microvilli, condensed or fragmented nuclei, and the formation of membrane vesicles, as in etoposide (Fig. 4). Cells treated with Pep27anal2 increased apoptosis significantly (*P *< 0.05) in a time-dependent manner. Moreover, 62.5 μM Pep27anal2 induced more apoptosis than 100 μM of etoposide (Fig. 4).

### Pep27anal2 does not induce cytochrome c release from mitochondria to the cytosol in Jurkat cells

**Figure 5:**
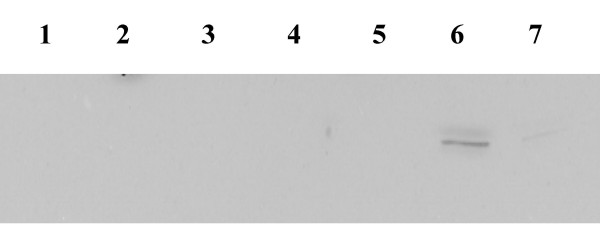
**Western blotting of cytochrome c released from mitochondria. **Jurkat cells (5 × 10^5^/ml) were treated with Pep27anal2 at 12.5 μM for 1 h, 2 h, and 4 h, and at 62.5 μM for 4 h. Mitochondria were pelleted by centrifugation, and the supernatants retained. As a control, mitochondria were treated with 0.1% (vol/vol) SDS, which induces cytochrome c release from mitochondria. Protein from each fraction was subjected to SDS/PAGE for Western blot analysis using an anti-cytochrome c antibody. 1, the result of analyzing 50 μg of cytosol after adding no Pep27anal2; 2, 50 μg cytosol after 12.5 μM for 1 hr; 3, 50 μg cytosol after 12.5 μM Pep27anal2 for 2 hr; 4, 50 μg cytosol after 12.5 μM Pep27anal2 for 4 hr; 5, 50 μg cytosol after 62.5 μM Pep27anal2 for 4 hr; 6, 50 μg mitosol after 0.1% SDS; 7, 25 μg mitosol after 0.1% SDS.

Cytochrome c is a well-characterized mitochondrial protein and is involved in cellular energy metabolism. The precursor of cytochrome c, apocytochrome c, is synthesized in the cytoplasm. Upon translocation to mitochoindria, cytochrome c is refolded and acquires a heme moiety, which is required for functionality in the mitochondrial respiration chain. The heme-bound form of cytochrome c is called holocytochrome c, which can activate the caspases responsible for apoptosis by interacting with protease activating factors when released into the cytosol [17]. Jurkat cells (5 × 10^5^/ml) were treated with Pep27anal2 at a concentration of 12.5 μM for 1 h, 2 h, and 4 h and at 62.5 μM for 4 h. After these incubations, cytochrome c in the cytosol fraction, obtained by 14,000 g centrifugation for 15 min, and in the mitosol, obtained by adding 0.1% SDS to the mitochondrial fraction, was determined by Western blotting. Pep27anal2 at 62.5 μM did not induce cytochrome c release from mitochondria into the cytosol within 4 hrs (Fig. 5).

### Solution structures of both Pep27 and Pep27anal2

**Figure 6:**
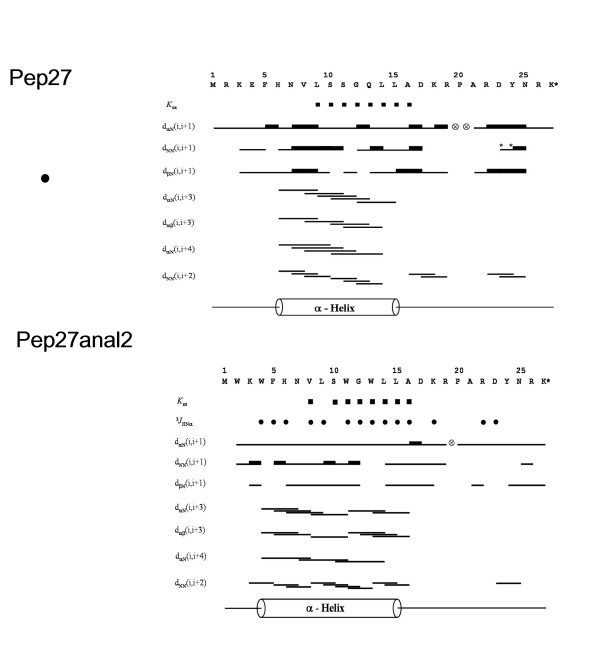
**Summary of the sequential and short-range NOEs for Pep27 and for Pep27anal 2 in TFE/water (1:1 v/v) at pH 7.2 and 15°C, showing sequential and short-range NOE contacts**. Amide proton exchange rates (■), backbone vicinal coupling constants (●; ^3^*J*_HNα _< 6 Hz) are also indicated. represents a proline residue The helical region is represented as a long cylinder.

**Figure 7:**
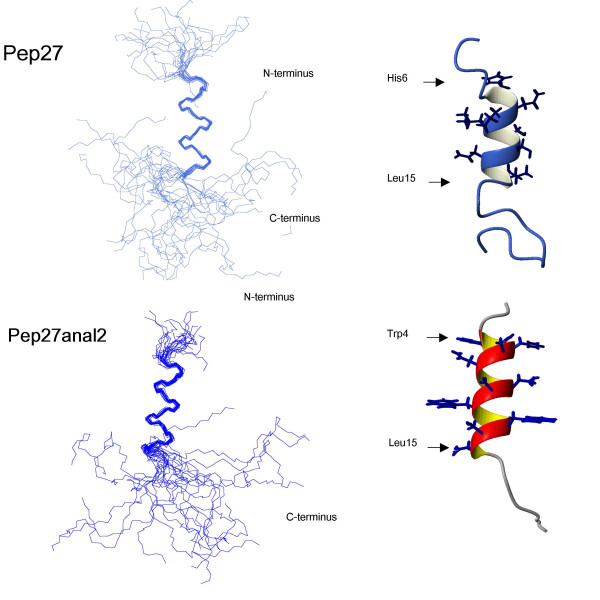
**A comparison of the 3D solution structures of Pep27 and Pep27anal2. **Final simulated-annealing structures of Pep27 and Pep27anal2. Superposition of the final 20 <SA>_k _structures of Pep27 and Pep27anal2 over the energy-minimized average structure () is shown. Backbone atoms in Heavy backbone atoms are superimposed for Pep27 His6-Leu15 and Pep27anal 2 residues Trp4-Leu15. The helical ribbon was generated using the MOLMOL program.

Complete proton resonance assignments for Pep27 and Pep27anal2 were obtained using the standard sequential resonance assignment procedure. Once the individual spin systems had been classified, backbone sequential resonance assignments were completed by *d*_αN _(*i*,*i*+1) NOE connectivities in the 2D-NOESY spectra. Figure 6 summarizes the sequential and short-range NOE connectivities observed for Pep27 and Pep27anal2 in TFE/H_2_O (1v/1v). The observations of continuous *d*_NN_(*i*,*i*+1) contacts, together with the characteristic *d*_αN_(*i*,*i*+3) and *d*_αβ _(*i*,*i*+3) NOEs strongly support the existence of α-helices (Fig. 6). The amide proton temperature coefficient also supports the hypothesis that intramolecular hydrogen bonds stabilize the structures of the Pep27 and Pep27anal2 helices. This result is further supported by the backbone amide proton exchange data of both peptides [18], and the small ^3^*J*_HNα _coupling constants of Pep27anal2 (that of Pep27 could not be measured). The NMR structures of Pep27 and Pep27anal2 were calculated using the experimental restraints derived from the 2D-NOESY spectra. A total of 50 distance geometry geometric structures served as starting structures for the dynamic simulated-annealing calculations for the peptides in TFE/H_2_O solution. The 20 lowest-energy structures (<SA>_k_) of 50 simulated annealing structures were selected for detailed structural analysis. A best-fit superposition of 20 <SA>_k _structures and an energy-minimized average structure () are shown in Fig. 7. The main secondary-structural feature of Pep27 is one α-helix spanning residues, His6 -Leu15 in TFE/H_2_O (1v/1v), whereas Pep27anal2 has a more stable α-helix spanning Trp 4-Leu15 (Fig. 7).

## Discussion

In this study, we investigated the anticancer activities of Pep27 analogues. Although Pep27 did not show anticancer activity at concentrations greater than 70 μM, its analogues were found to have various cytotoxicities in a number of cancer cell lines. Of the five Pep27 analogue peptides synthesized, Pep27anal2 showed both greatest hydrophobicity and anticancer activity. Our results suggest that substitutions of (^2^R→W), (^4^E→W), (^11^S→W), and (^13^Q→W) in Pep27 anal2 plays an important role in its hydrophobicity and/or anticancer activity. In particular, the latter two substitutions contribute hydrophobicity or anticancer activity to Pep27anal2, by virtue of the lower anticancer activity of Pep27anal1, containing (^2^R→W) and (^4^E→W) substitutions. In addition, substitutions with the more hydrophobic amino acid tryptophan were not always reflected by a higher hydrophobicity or a greater anticancer activity. Pep27anal2 had greater hydrophobicity than other analogues as assessed by RP-HPLC. A positive linear correlation was observed between apoprotein hydrophobicity and column retention time obtained by RP-HPLC [19]. Thus, it could be implicated that the hydrophobicity of peptides, rather than the number of hydrophobic amino acids, may play an important role in anticancer activity or membrane transport. Since peptides cannot readily enter cells, substitution of amino acids not interfering with activity or conjugation with other peptides to facilitate peptide cell entry has been tried. Certain short peptides, which are able to traverse the cell membranes, and which have low lytic activity, can be useful as carriers (vectors) for hydrophilic molecules. Cell penetrating peptides include pAntp ('penetratin'), transportan, MAP (KLAL), Tat (48–60) and VP22. pAntp is a 60-amino acid polypeptide with a sequence that corresponds to the *Drosophila antennapedia *homeobox gene [20]. Transportan is a 27 amino acid peptide containing 12 functional amino acids from the amino terminus of the neuropeptide galanin and mastoparan at the carboxyl terminus, which are connected by a lysine [21]. MAP (KLAL peptide) is derived from an amphipathic model peptide, KLALKLALKALKAAKLA-NH2 [22]. TAT protein from human immunodeficiency virus (HIV-1), is able to deliver biologically active proteins in vivo, and is of considerable interest for protein therapeutics [23]. Recently the HIV-1 transcription (Tat) protein transduction domain (RKKRRQRRR) has been conjugated into various types of amino acids or proteins [24-26]. Of the cell penetrating peptides, MAP (KLAL) was found to have the fastest uptake, followed by transportan, Tat(48–60), and finally, penetratin [27]. The herpes simplex virus type 1 tegument protein VP22 has recently been shown to mediate the intercellular transport of proteins, raising the possibility that it may be helpful in a setting where the global delivery of therapeutic proteins is desired [28,29].

In the present study, confocal microscopy showed that Pep27anal2 does not act as a membrane toxin like mellitin, but rather penetrates cells by an unknown mechanism, and induces apoptosis as characterized by cell shrinkage and membrane blebbing. Flow cytometry was performed to determine if Pep27anal2 induces apoptosis and membrane damage. Annexin V binding with the cell surface was used as being indicative of apoptosis, in conjunction with a dye exclusion test to establish integrity of the cell membrane. Annexin V is a Ca^2+ ^dependent phospholipid-binding protein with high affinity for phosphatidylserine. However, the translocation of phosphatidylserine to the external cell surface is not unique to apoptosis, and also occurs during necrosis. The difference between these two forms of cell death is that during the initial stages of apoptosis the cell membrane remains intact, while at the very moment that necrosis occurs the cell membrane looses its integrity, becoming leaky [16]. Pep27anal2 increased the annexin V-FITC staining in Jurkat cells, whereas mellitin at the same concentration highly increased the distribution of propidium iodide staining. However, much higher concentrations (62.5 μM) of Pep27anal2 further distribution of annexin V-FITC staining, whereas propidium iodide staining increased only slightly. Cell-penetrating peptides generally have the ability to gain cell entry in an energy-dependent manner [26]. In the present study, Pep27anal2-induced apoptosis was not influenced by pretreating with the ATP depleter sodium azide (data not shown), suggesting an energy-independent transport and/or apoptosis mediated by Pep27anal2. In addition, electron microscopy revealed that Pep27anal2 induced the morphological features of apoptosis in Jurkat cells, and showed cytoplasmic condensation, cell shrinkage, loss of plasma membrane microvilli, condensed or fragmented nuclei, and the formation of membrane vesicles. The highest apoptotic indices were observed for Pep27anal2, and this was statistically significant. Cells undergoing apoptosis *in vivo *showed increased cytochrome c release to the cytosol, suggesting that mitochondria may be involved by releasing cytochrome c [30]. When cytochrome c is released into the cytosol, it activates the caspases responsible for apoptosis by interacting with protease activating factors [15]. In the cytosol, cytochrome c binds to Apaf-1, which triggers the activation of caspase-9. Caspase-9 is believed to propagate the death signal by triggering six additional caspases (caspases-2, -3, -6, -7, -8, and -10) but not caspases-1, -4, and -5 [31]. However, Pep27anal2 did not appear to induce cytochrome c release from mitochondria within 4 h, suggesting that it induces apoptosis by a cytochrome c-independent mechanism. In addition, the anticancer activity of Pep27anal2 was not abrogated by the pan-caspase inhibitor Z-VAD-fmk (data not shown), indicating that Pep27anal2-induced apoptosis proceeds via a caspase-independent pathway. Like Pep27anal2, there have been reported about apoptosis induced in a caspase-independent fashion without subsequent release of cytochrome c [32,33].

Based on the elucidated solution structures, Pep27 has a short coil-helix-coil composed of residues 6–16. However, the Pep27anal2 analogue characterized by substitutions at ^2^R→W, ^4^E→W, ^11^S→W and ^13^Q→W was found to have a slightly longer more stable helical conformation than Pep27. Our biological activity data also showed that Pep27anal2 is more potent than Pep27. This may have been due to an increased hydrophobic interaction by Pep27anal2. Therefore, we suggest that helical stability has an important role in the biological activity of Pep27. Taken together, our results indicate that the increased hydrophobicity of Pep27anal2 aids its penetration of the membrane. This is followed by apoptotic induction, but which does not involve cytochrome c release from mitochondria. The hydrophobicity of Pep27anal2 appears to play an important role in its ability to penetrate the plasma membrane and/or in its anticancer activity. Thus, we believe that Pep27anal2 is a potential candidate for the design of anticancer peptides.

## Abbreviations

RP, reverse phase ; MALDI, matrix-assisted laser desorption ionization; PI, propidium iodide; DSS, sodium 2, 2-dimethyl-2-silapentane-5-sulfonic acid; MTT, 3-(4,5-dimethylthiazol-2-yl)-2,5-diphenyl tetrazolium bromide; TFE, 2, 2, 2-trifluoro-(d_3_)-ethanol; PBS, phosphate-buffered saline; TBS, Tris-buffered saline; SDS, sodium dodesyl sulfate
